# Biomarkers of Extracellular Matrix Metabolism (MMP-9 and TIMP-1) and Risk of Stroke, Myocardial Infarction, and Cause-Specific Mortality: Cohort Study

**DOI:** 10.1371/journal.pone.0016185

**Published:** 2011-01-19

**Authors:** Jonas Hansson, Ramachandran S. Vasan, Johan Ärnlöv, Erik Ingelsson, Lars Lind, Anders Larsson, Karl Michaëlsson, Johan Sundström

**Affiliations:** 1 Department of Medical Sciences, Uppsala University, Uppsala, Sweden; 2 Framingham Heart Study, Framingham, Massachusetts, United States of America; 3 Sections of Cardiology and Preventive Medicine, Boston Medical Center, Boston University School of Medicine, Boston, Massachusetts, United States of America; 4 Department of Public Health and Caring Sciences, Uppsala University, Uppsala, Sweden; 5 School of Health and Social Sciences, Dalarna University, Falun, Sweden; 6 Department of Medical Epidemiology and Biostatistics, Karolinska Institutet, Stockholm, Sweden; 7 Department of Surgical Sciences, Uppsala University, Uppsala, Sweden; 8 Uppsala Clinical Research Center, Uppsala University, Uppsala, Sweden; Universitätsklinikum Schleswig-Holstein, Germany

## Abstract

**Objective:**

Turnover of the extracellular matrix in all solid organs is governed mainly by a balance between the degrading matrix metalloproteinases (MMPs) and their tissue inhibitors (TIMPs). An altered extracellular matrix metabolism has been implicated in a variety of diseases. We investigated relations of serum levels of MMP-9 and TIMP-1 to mortality risk from an etiological perspective.

**Design:**

The prospective Uppsala Longitudinal Study of Adult Men (ULSAM) cohort, followed from 1991–1995 for up to 18.1 years. A random population-based sample of 1,082 71-year-old men, no loss to follow-up. Endpoints were all-cause (n = 628), cardiovascular (n = 230), non-cardiovascular (n = 398) and cancer mortality (n = 178), and fatal or non-fatal myocardial infarction (n = 138) or stroke (n = 163).

**Results:**

Serum MMP-9 and TIMP-1 levels were associated with risk of all-cause mortality (Cox proportional hazard ratio [HR] per standard deviation 1.10, 95% confidence interval [CI] 1.03–1.19; and 1.11, 1.02–1.20; respectively). TIMP-1 levels were mainly related to risks of cardiovascular mortality and stroke (HR per standard deviation 1.22, 95% CI 1.09–1.37; and 1.18, 1.04–1.35; respectively). All relations except those of TIMP-1 to stroke risk were attenuated by adjustment for cardiovascular disease risk factors. Relations in a subsample without cardiovascular disease or cancer were similar to those in the total sample.

**Conclusion:**

In this community-based cohort of elderly men, serum MMP-9 and TIMP-1 levels were related to mortality risk. An altered extracellular matrix metabolism may be involved in several detrimental pathways, and circulating MMP-9 or TIMP-1 levels may be relevant markers thereof.

## Introduction

In every part of the human body, the extracellular matrix plays an important part as the framework of the parenchymal tissue. An abnormal extracellular matrix regulation has been implicated as a direct causal factor in a number of important disease processes, such as cancer invasion and metastasis,[1] atherosclerotic plaque rupture,[2] congestive heart failure development[3] and alveolar wall destruction in chronic obstructive pulmonary disease.[4] The metabolism of the extracellular matrix is governed by a balance between the degrading matrix metalloproteinases (MMPs) and their tissue inhibitors (TIMPs).[5] Some of these matrix markers are possible to measure in serum or plasma, thus the possibility of monitoring matrix turnover by means of simple blood tests is an alluring concept.

Two of the matrix markers most consistently indicted in cardiovascular disease development and prognosis are MMP-9 and TIMP-1.[6] Circulating levels of these have been related to most cardiovascular disease risk factors in large community-based samples[7], [8], [9], [10] and have been associated with risk of death in patients with known cardiovascular disease.[11], [12], [13] In studies of limited sample sizes, higher circulating MMP-9 and TIMP-1 levels have also indicated worse prognosis in several cancer types, including lung[14] and breast cancer,[15] and higher circulating TIMP-1 levels have also portended worse prognosis in colorectal[16] and gastric cancer.[17]

Relations of circulating MMP-9 and TIMP-1 levels to mortality risk in the general population are little known. We aimed to study relations of MMP-9 and TIMP-1 levels to risk of cause-specific mortality and cardiovascular events from an etiological perspective, in a well-characterized community-based cohort of elderly men.

## Methods

### Ethics Statement

All participants gave written consent, and the Uppsala University ethics committee approved the study.

### Study Sample

We used the Uppsala Longitudinal Study of Adult Men (ULSAM), a prospective cohort study to which all 50-year-old men living in Uppsala County, Sweden, in 1970–1973 were invited (www.pubcare.uu.se/ULSAM). Out of 2,841 invited, 2,322 accepted. The cohort was re-examined at circa age 60 and age 71, the latter examination (in 1991–1995) defining the baseline cohort of the present study. Of the 1,681 71-year-old men alive, 1,221 (73%) participated. A further 100 men were excluded because of lack of matrix biomarker measurements, and another 39 due to lacking covariates, rendering a sample of 1,082 men eligible for the present study. A subsample of 818 men without cardiovascular disease (myocardial infarction [ICD-9 code 410, ICD-10 code I21], stroke [ICD-9 codes 430–433 & 436; ICD-10 codes I60–I64], heart failure [chart review-validated cases], or electrocardiographic Q-waves or left bundle-branch block [Minnesota codes 1.1 and 7.1]) or cancer (ICD-9 codes 140–209; ICD-10 codes C00–C99) was also investigated in order to minimize influence of reverse causation.

### Baseline Investigations

At the age 71 examination, all participants answered self-administered questionnaires, underwent a medical examination, blood sampling after an overnight fast, supine blood pressure measurements, and anthropometric measurements as described previously. [18]

Smoking experience was classified into four levels: never smoking, current smoking, or two levels of previous smoking (above or below median [33] of pack-years of previous smoking). Diabetes at baseline was defined as fasting plasma glucose > = 7.0 mmol/L or use of oral hypoglycemic agents or insulin. Office systolic and diastolic blood pressures were measured twice in the right arm to the nearest even number with the subject in the supine position after resting for 10 minutes, and the means of the two measurements were used. Use of antihypertensive or lipid-lowering drugs was classified using questionnaires. Cholesterol concentration was analyzed by an enzymatic technique. High sensitivity C-reactive protein and cystatin C were measured using latex enhanced reagent (Dade Behring, Deerfield, IL, USA) using a Behring BN ProSpec analyzer (Dade Behring). Glomerular filtration rate was estimated from serum cystatin C by the formula eGFR  = 77.24*[cystatin C]-1.2623.

MMP-9 and TIMP-1 were measured in fasting serum samples frozen shortly after sampling and then stored in -70 degrees C awaiting analysis. The samples were thawed and analyzed in early 2007 using commercial ELISA assays provided by R&D Systems Europe Ltd (Abingdon, United Kingdom). The samples had when thawed been frozen for a mean of 13.5 years (extremes 11.6–15.4 years), herein denoted “freezer time”.

### Follow-Up and Outcome Parameters

For the primary outcome, all-cause mortality, participants were followed from the baseline examination in 1991–1995 to October 4^th^, 2009, with a maximum of 18.1 years of follow-up (median 14.7 years; 13,682 person-years at risk). For all other outcomes, follow-up data was available until December 31, 2007, rendering up to 16.4 years of follow-up. We investigated six outcomes: all-cause mortality; cardiovascular mortality (ICD-9 codes 390–459; ICD-10 codes I00-I99); non-cardiovascular mortality (all other deaths); cancer mortality (ICD-9 codes 140–209; ICD-10 codes C00-C99); fatal or non-fatal myocardial infarction (ICD-9 code 410, ICD-10 code I21); and fatal or non-fatal stroke (ICD-9 codes 430–433 & 436; ICD-10 codes I60–I64). The primary outcome was defined using the Swedish census registry, which is updated within a few days after a person's death. All other outcomes were defined using the Swedish national Cause-of-Death and In-Patient registries, which are updated yearly with some lag. All three data sources include all Swedish citizens, and none of the study participants emigrated during follow-up; therefore we had zero loss to follow-up.

### Statistical Analysis

Initially, distributional properties of all variables were examined (Figures S1 and S2). We thereafter examined relations of MMP-9 and TIMP-1 to the six outcomes (all-cause mortality, CVD mortality, non-CVD mortality, cancer mortality, fatal or non-fatal myocardial infarction, and fatal or non-fatal stroke), each matrix biomarker and outcome in a separate set of models, using Cox proportional hazards analyses.

We investigated relations per standard deviation (SD) and by quartiles of MMP-9 and TIMP-1 to all outcomes. Freezer time was a priori decided upon as a covariate in all models, as the effects of long-term freezing on these biomarkers is not known. Both MMP-9 (r = 0.07, p = 0.02) and TIMP-1 (r = −0.10, p = 0.002) levels were statistically significantly albeit weakly related to freezer time. Age was adjusted for in all models by using age as the timeline. We considered three sets of models, in a hierarchical fashion: A) models adjusted for age and freezer time; B) models additionally adjusted for systolic blood pressure, antihypertensive treatment, lipid-lowering treatment, diabetes mellitus, body mass index, total cholesterol, estimated glomerular filtration rate, and smoking status; and C) models additionally adjusted for C-reactive protein (because inflammation has been identified as an important MMP stimulus). Model A is the primary model, and models B and C are viewed as mechanistic models – if substantial parts of the effects of cardiovascular disease risk factors or inflammation on risk are mediated by matrix remodeling (as reflected in altered levels of matrix markers), the matrix marker estimates will be lower in these models than in models A.

Schoenfeld's tests and inspection of Schoenfeld residuals and Nelson-Aalen curves were used to confirm proportionality of hazards, and Martingale residuals and multivariable-regression spline models with 5 degrees of freedom for the matrix markers were inspected to rule out deviations from linearity, in models A and B. We investigated additive interactions between the matrix markers (4^th^ vs. 1^st^ quartiles) and the covariates by calculating the relative excess risk due to interaction (RERI),[19] and multiplicative interactions between the matrix markers (on their continuous scale) and the covariates were tested using product terms. Stata 10.1 was used for all calculations.

## Results

Clinical characteristics of the study sample at baseline are presented in Table 1.

**Table 1:** Characteristics at Baseline.

	Total Sample (n = 1,082)	Subsample without CVD or cancer (n = 818)
*Clinical Variables*		
Age, years	71.0 (0.6)	71.0 (0.6)
Diabetes mellitus	188 (17.3)	131 (16.0)
Hypertension	807 (74.6)	600 (73.4)
Systolic blood pressure, mmHg	147 (18)	148 (19)
Diastolic blood pressure, mmHg	84 (10)	84 (9)
Antihypertensive treatment	387 (35.8)	249 (30.4)
Current smoking	236 (21.8)	179 (21.9)
Never-smoking	335 (31.0)	249 (30.4)
Low previous smoking	377 (34.8)	226 (27.6)
High previous smoking	370 (34.2)	164 (20.1)
Waist circumference, cm	94.7 (9.6)	94.4 (9.5)
Body mass index, kg/m^2^	26.2 (3.4)	26.1 (3.4)
s-Cholesterol, mmol/L	5.80 (0.98)	5.79 (0.99)
s-HDL-cholesterol, mmol/L	1.28 (0.35)	1.30 (0.35)
s-Triglycerides, mmol/L	1.28 (0.78)	1.22 (0.79)
Lipid lowering treatment	100 (9.7)	66 (8.5)
Estimated glomerular filtration rate, mL/min	74.3 (14.8)	93.4 (19.0)
s-C-reactive protein, mg/L	3.4 (4.9)	3.3 (4.7)
*Matrix Biomarkers*		
s-MMP-9, ng/L	332 (234)	332 (236)
s-TIMP-1, ng/L	200 (71)	199 (73)

Data are numbers (percent) or means (standard deviations), and medians (interquartile ranges) for triglycerides, MMP-9 and TIMP-1. HDL, high-density lipoprotein; MMP-9, matrix metalloproteinase-9; TIMP-1, tissue inhibitor of metalloproteinases-1.

During follow-up for all-cause mortality, 628 of the 1,082 men in the total sample died (rate 45.9/1,000 person-years at risk). During follow-up for cause-specific mortality and cardiovascular events, 230 died from cardiovascular disease and 178 of cancer (Table 2).

**Table 2:** Numbers of Events and Incidence Rates.

Outcome	Person-years at risk	Number of events	Incidence rate (95% confidence interval) per 1,000 person-years at risk
*Total Sample (n = 1,082)*			
All-cause mortality	13,682	628	45.9 (42.4–49.6)
CVD mortality	12,792	230	18.0 (15.8–20.5)
Non-CVD-mortality	12,792	398	31.1 (28.2–34.3)
Cancer mortality	12,792	178	13.9 (12.0–16.1)
Myocardial infarction	11,272	138	12.2 (10.4–14.5)
Stroke	11,802	163	13.8 (11.8–16.1)
*Subsample without CVD or cancer (n = 818)*			
All-cause mortality	10,746	435	40.5 (36.8–44.5)
CVD mortality	10,000	151	15.1 (12.9–17.7)
Non-CVD-mortality	10,000	284	28.4 (25.3–31.9)
Cancer mortality	10,000	120	12.0 (10.0–14.4)
Myocardial infarction	9,547	107	11.2 (9.3–13.5)
Stroke	9,529	122	12.8 (10.7–15.3)

CVD, cardiovascular disease.

**Figure 1:**
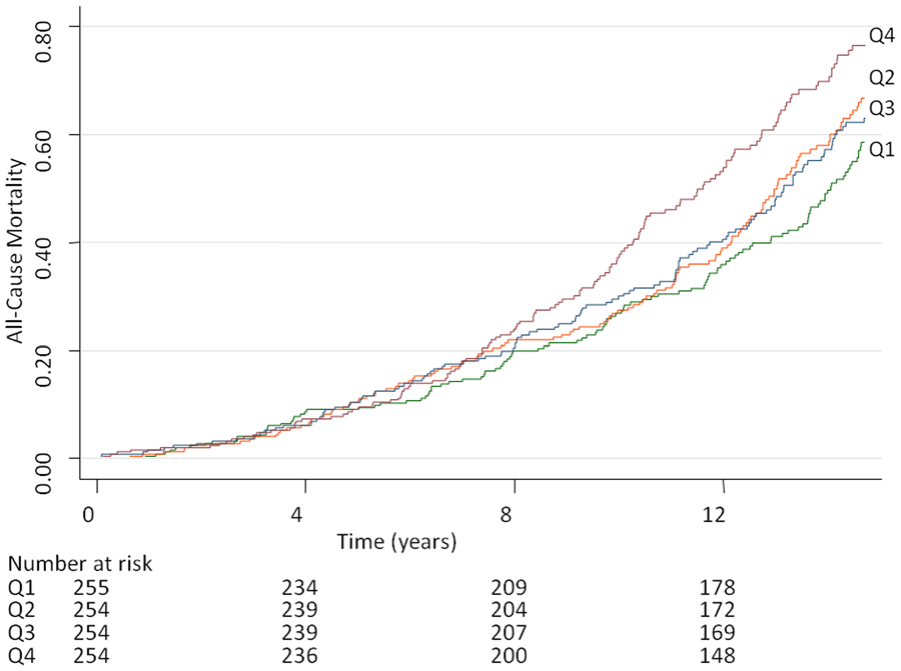
All-Cause Mortality by Quartiles of s-MMP-9. Quartile (Q) limits for matrix metalloproteinase-9 (MMP-9) were 228, 332 and 462 ng/L.

**Figure 2:**
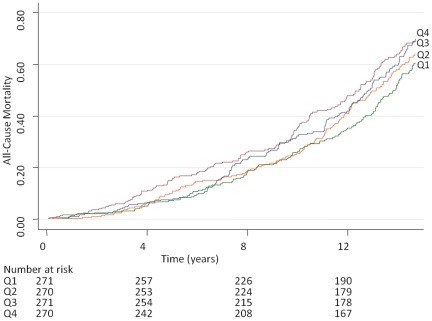
All-Cause Mortality by Quartiles of s-TIMP-1. Quartile (Q) limits for tissue inhibitor of metalloproteinases-1 (TIMP-1) were 166, 200 and 238 ng/L.

Cumulative incidence of all-cause mortality by quartiles of MMP-9 and TIMP-1 in the total sample is displayed in Figures 1 and 2. Relations of both MMP-9 and TIMP-1 to all-cause mortality were observed (Table 3). The notion of essentially linear relations was supported by analyses by quartiles of MMP-9 and TIMP-1 in Figures 1 and 2 and Figure S3 (and was confirmed by spline analyses and inspection of Martingale residuals).

**Table 3:** Relations of Matrix Biomarkers to Risk of Cause-Specific Mortality and Cardiovascular Events.

	Matrix metalloproteinase-9						Tissue inhibitor of metalloproteinases-1					
	Models A		Models B		Models C		Models A		Models B		Models C	
*Total Sample (n = 1,082)*												
All-cause mortality	1.10	(1.03–1.19)	1.03	(0.95–1.12)	1.00	(0.91–1.08)	1.11	(1.02–1.20)	1.09	(1.00–1.19)	1.06	(0.97–1.17)
CVD mortality	1.09	(0.97–1.23)	0.99	(0.87–1.13)	0.97	(0.84–1.11)	1.22	(1.09–1.37)	1.13	(0.98–1.30)	1.10	(0.96–1.27)
Non-CVD mortality	1.11	(1.02–1.22)	1.06	(0.96–1.18)	1.02	(0.92–1.14)	1.04	(0.93–1.16)	1.07	(0.95–1.20)	1.05	(0.93–1.18)
Cancer mortality	1.08	(0.94–1.24)	1.02	(0.87–1.19)	0.95	(0.80–1.13)	1.10	(0.95–1.27)	1.15	(0.98–1.34)	1.11	(0.94–1.30)
Myocardial infarction	0.98	(0.83–1.17)	0.91	(0.76–1.09)	0.88	(0.72–1.06)	1.14	(0.98–1.33)	1.17	(0.98–1.39)	1.14	(0.96–1.36)
Stroke	1.00	(0.86–1.17)	0.95	(0.80–1.12)	0.92	(0.77–1.10)	1.18	(1.04–1.35)	1.20	(1.03–1.39)	1.18	(1.01–1.38)
*Subsample without CVD or cancer (n = 818)*												
All-cause mortality	1.14	(1.04–1.25)	1.07	(0.97–1.18)	1.04	(0.94–1.16)	1.10	(1.00–1.21)	1.11	(1.00–1.24)	1.09	(0.98–1.22)
CVD mortality	1.14	(0.97–1.33)	1.02	(0.86–1.21)	1.00	(0.84–1.20)	1.25	(1.10–1.42)	1.21	(1.03–1.42)	1.19	(1.00–1.40)
Non-CVD mortality	1.14	(1.02–1.28)	1.11	(0.98–1.26)	1.07	(0.94–1.23)	1.02	(0.89–1.16)	1.07	(0.93–1.23)	1.05	(0.90–1.21)
Cancer mortality	1.13	(0.95–1.34)	1.07	(0.88–1.31)	1.01	(0.82–1.26)	1.10	(0.93–1.31)	1.18	(0.98–1.43)	1.15	(0.94–1.40)
Myocardial infarction	1.06	(0.87–1.28)	0.97	(0.79–1.19)	0.94	(0.76–1.17)	1.16	(0.98–1.37)	1.20	(0.99–1.45)	1.18	(0.97–1.44)
Stroke	0.91	(0.74–1.12)	0.85	(0.68–1.06)	0.81	(0.64–1.03)	1.20	(1.03–1.38)	1.23	(1.05–1.44)	1.21	(1.03–1.43)

Data are Cox proportional hazard ratios (95% confidence intervals) per 1-standard deviation of the matrix biomarker. Models A adjusted for age and freezer time; models B additionally adjusted for systolic blood pressure, antihypertensive treatment, lipid-lowering treatment, diabetes mellitus, body mass index, total cholesterol, estimated glomerular filtration rate, and smoking status; models C additionally adjusted for C-reactive protein. CVD, cardiovascular disease.

The relation of MMP-9 level to all-cause mortality risk appeared to be driven by a relation to non-cardiovascular mortality risk, but not to cancer mortality risk. These relations were attenuated upon adjustment for covariates in models B and C (Table 3).

The relation of TIMP-1 level to all-cause mortality risk was driven by a relation to cardiovascular mortality risk. The relation to all-cause mortality risk was slightly attenuated by adjustment for covariates in model B, and more so by further adjustment for C-reactive protein. TIMP-1 level was most robustly related to risk of stroke. The relation to stroke risk was not affected by adjustment for covariates, including C-reactive protein (Table 3).

Relations of matrix biomarkers to outcomes in the subsample without cardiovascular disease or cancer were similar to those in the total sample (Table 3). Relations of TIMP-1 level to risks of cardiovascular mortality and stroke were slightly stronger in this subsample than in the total sample. The relation to cardiovascular mortality risk was less affected by adjustment for covariates in the subsample than in the total sample (Table 3).

Assumptions of proportionality of hazards were met. No interactions with any of the covariates were observed. Relations of covariates to risk of outcomes in the total sample are displayed in Table S1.

## Discussion

The primary observation in this community-based sample of men was that higher levels of circulating MMP-9 or TIMP-1 were associated with higher risk of death. Higher TIMP-1 levels were mainly related to higher risk of stroke and cardiovascular mortality, and higher MMP-9 levels mainly to risk of non-cardiovascular mortality. The observed relations were of modest magnitude, and the relations of TIMP-1 to stroke risk were not explained to any major extent by established cardiovascular disease risk factors or C-reactive protein.

### Comparisons with Previous Studies

Earlier studies of associations of levels of circulating biomarkers of extracellular matrix turnover with mortality risk have so far mainly been made in groups of patients with manifest cardiovascular disease or cancer. In previous studies of patients with atherosclerotic cardiovascular disease, TIMP-1 has been observed to predict cardiovascular[11], [12] and all-cause mortality[11] and cardiovascular events,[20] and MMP-9 has been associated with risk of cardiovascular death in one study[13] and a combined endpoint of stroke or cardiovascular death in another,[21] but no relation was observed in one study.[11] In one study of men, TIMP-1 was not related to prognosis,[22] and in another population-based study selected based on left ventricular geometry and with limited number of cases, TIMP-1 was related to mortality risk but not risk of cardiovascular events, and MMP-9 measured with an assay with limited sensitivity was not related to any outcome.[23] Studies in samples of heart failure patients have been inconsistent, with reports of relations of these markers with prognosis in some[24] but not other studies.[25] MMP-9 level has been related to more severe neurological deficits[26] and increased risk of hemorrhagic transformation of infarcts in stroke patients[27], [28], [29], but relations of TIMP-1 to stroke outcomes are unknown. Relations of these markers to incident stroke risk in the general population are also unknown. In one recent large nested case-control study, relations of MMP-9 to myocardial infarction and stroke were observed in unadjusted models, but not in multivariable-adjusted ones.[30]

The present study is in line with previous observations in patient samples of associations of TIMP-1 with all-cause and cardiovascular mortality, but extends these to a population-based setting and identifies a robust relation to stroke risk. The weaker associations of MMP-9 than TIMP-1 levels to cardiovascular mortality in the present study are also in line with the previous literature, but are now demonstrated in the general population.

Results of studies in small samples of cancer patients have been inconsistent, but higher circulating MMP-9 and TIMP-1 levels have indicated worse prognosis in studies of several cancer types.[14], [15], [16], [17] No previous studies have reported associations of levels of matrix biomarkers to cancer incidence. The present study does not support strong such associations for major cancer types, but cannot exclude modest relations or relations to less frequent cancer types.

### Potential Mechanisms

Relations of TIMP-1 to cardiovascular disease risk may have a variety of explanations. TIMP-1 is the main inhibitor of MMP-9, but also inhibits several other MMPs. Discrepant findings between MMP-9 and TIMP-1 is therefore not unexpected.

The investigated proteins may be causally involved in atherosclerosis development. In experimental settings, increased TIMP-1 expression has led to decreased atherosclerosis development in some[31]–[32] but not other studies,[33] and decreased MMP-9 expression has also led to a reduced atherosclerotic burden.[34]

MMP-9 and TIMP-1 may also be causally involved in cardiac dysfunction. In animal models of heart failure of different origin, pharmacological MMP inhibition reduces left ventricular dilatation and preserves cardiac systolic function[35], [36] and transgenic animal models also support the notion of detrimental effects of MMP-9 and beneficial effects of TIMP-1 after myocardial infarction.[37], [38] In general population-based samples, circulating MMP-9 and TIMP-1 levels have been related to left ventricular hypertrophy,[7], [8], [9] and TIMP-1 levels inversely to left ventricular systolic function.[7], [8] One mechanism behind the relations to heart failure development may be intracellular cleaving of myosin filaments by MMP-9.[39] The sources of the matrix markers in this study are unknown, but relations of circulating and myocardial levels of some matrix markers are substantial.[6]

One explanation behind the observed robust relations of TIMP-1 to stroke risk may be that higher TIMP-1 levels signify higher left ventricular mass.[7], [8] Left ventricular hypertrophy is a powerful risk factor for stroke, possibly because it may lead to atrial fibrillation.

Explanations behind the observed relation of MMP-9 to non-cardiovascular death are less substantiated. The matrix markers were not important risk factors for cancer mortality in the present study, but previous associations of these markers with prognosis in patients with malignant disease have been explained by an MMP-independent growth-promoting capacity of TIMP-1,[40] and effects of MMP-9 on tumor growth, metastasis and angiogenesis.[41]

MMP-9 has been demonstrated to regulate leukocyte function through a number of mechanisms, including activation of pro-IL-1β into IL-1β and truncation of IL-8 into a more active form and, as degranulation of MMP-9 from neutrophils is stimulated by IL-8, thus creating a positive feedback loop.[42] MMP-9 may therefore potentially play a role in the course of infectious diseases; and possibly in pulmonary disease.

Altered extracellular matrix turnover is a known cause of osteoporosis and fractures, which in some cases can lead to fatalities. This could also explain part of the non-cardiovascular mortality.

All relations of MMP-9 to outcomes were attenuated by adjustment for covariates, which is consistent with the notion that MMP-9 levels may be on a causal pathway between the covariates and the outcomes. Relations of TIMP-1 level to stroke risk were independent of these covariates. The discrepancy between relations of MMP-9 and TIMP-1 to cardiovascular events was not just due to differences in measurement precision, as the point estimates were lower for MMP-9 relations. The fact that TIMP-1 is the inhibitor also of other MMPs than MMP-9 (notably MMP-1), and these MMPs may also be important in the pathophysiology of cardiovascular disease, may partly explain why TIMP-1 was a stronger marker than MMP-9 of cardiovascular disease risk in this and other studies.

Inflammation is a known trigger of MMP expression and secretion. The observation that the relation of TIMP-1 to stroke risk was independent of C-reactive protein levels, as well as other cardiovascular risk factors, may indicate that extracellular matrix metabolism is an important process *per se*. Similarly, TIMP-1 predicted cardiovascular events independently of C-reactive protein, interleukin-6 and white cell count in another study of a patient sample.[20] Extracellular matrix metabolism should therefore be viewed as an entity separate from cytokine-mediated inflammation, and its best measurement and prognostic value determined.

### Strengths and Limitations

Strengths of this study include the prospective cohort design, the relatively large number of participants, and the general population sample. The most obvious limitation is that it only comprises elderly men. Generalizability to women, younger persons, and non-Caucasians is unknown. Possible pre-analytical errors of TIMPs and MMPs have been a matter of debate. Falsely elevated serum levels due to release from leukocytes and thrombocytes during handling have been suggested.[43] On the other hand, this release may be a physiologically important property. One study reported decreasing levels of MMP-9, but not TIMP-1, in plasma over time in spite of frozen storage,[44] but the effects of freezing on serum levels are unknown. In this study, we accounted for time in the freezer in all statistical analyses. Because a smorgasbord of different MMPs and TIMPs exist, other MMP and TIMP species than the ones studied may be important. The cause of death is based on national registry data, and misclassification between causes of death is possible, but likely random. The investigation of multiple outcomes and multiple models is motivated in an etiological analysis such as the present, but may increase the risk of type-I error.

### Conclusions

In this study, higher levels of circulating MMP-9 or TIMP-1 were associated with higher risk of death. In particular, higher TIMP-1 levels were related to higher risk of stroke and cardiovascular death.

An altered extracellular matrix metabolism is indicted in several detrimental pathways. Based on these observations, the most important of these pathways from a mortality perspective may be primarily cardiovascular disease-related ones. Circulating MMP-9 or TIMP-1 levels may be promising markers of those matrix metabolism alterations. We encourage investigation of these associations in other general population samples, and investigation of relations of other markers of extracellular matrix metabolism to hard endpoints in the general population. This is a novel group of mortality risk factors reflecting pathways for which development of drugs is ongoing.[45] Before such drugs can be useful in clinical practice, tools for identification of target groups and for monitoring treatment need to be developed. Our findings may be an important step in that direction.

## Supporting Information

Figure S1 **Distribution of s-MMP-9.**
(TIF)

Figure S2 **Distribution of s-TIMP-1.**
(TIF)

Figure S3 **Relations of Quartiles of Matrix Biomarkers to Risk of Cause-Specific Mortality and Cardiovascular Events in the Total Sample.** Boxes are Cox proportional hazard ratios, lines are 95% confidence intervals, from models A (adjusted for age and freezer time) for quartiles of matrix biomarkers vs. lowest quartile. Quartile limits for MMP-9 were 228, 332 and 462 ng/L and those for TIMP-1 were 166, 200 and 238 ng/L.
(TIF)

Table S1 **Relations of Covariates to Risk of Cause-Specific Mortality and Cardiovascular Events in the Total Sample.** Data are Cox proportional hazard ratios (95% confidence intervals) from age-adjusted analyses. Associations reported per standard deviation of continuous variables, and for presence vs. absence of dichotomous variables. Previous and current smoking categories compared to never-smokers.
(DOC)

## References

[1] Fong KM, Kida Y, Zimmerman PV, Smith PJ (1996). TIMP1 and adverse prognosis in non-small cell lung cancer.. Clin Cancer Res.

[2] Lijnen HR (2003). Metalloproteinases in development and progression of vascular disease.. Pathophysiol Haemost Thromb.

[3] Peterson JT, Hallak H, Johnson L, Li H, O'Brien PM (2001). Matrix metalloproteinase inhibition attenuates left ventricular remodeling and dysfunction in a rat model of progressive heart failure.. Circulation.

[4] Belvisi MG, Bottomley KM (2003). The role of matrix metalloproteinases (MMPs) in the pathophysiology of chronic obstructive pulmonary disease (COPD): a therapeutic role for inhibitors of MMPs?. Inflamm Res.

[5] Sternlicht MD, Werb Z (2001). How matrix metalloproteinases regulate cell behavior.. Annu Rev Cell Dev Biol.

[6] Sundstrom J, Vasan RS (2006). Circulating biomarkers of extracellular matrix remodeling and risk of atherosclerotic events.. Curr Opin Lipidol.

[7] Hansson J, Lind L, Hulthe J, Sundstrom J (2009). Relations of serum MMP-9 and TIMP-1 levels to left ventricular measures and cardiovascular risk factors: a population-based study.. Eur J Cardiovasc Prev Rehabil.

[8] Sundstrom J, Evans JC, Benjamin EJ, Levy D, Larson MG (2004). Relations of plasma total TIMP-1 levels to cardiovascular risk factors and echocardiographic measures: the Framingham heart study.. Eur Heart J.

[9] Sundstrom J, Evans JC, Benjamin EJ, Levy D, Larson MG (2004). Relations of plasma matrix metalloproteinase-9 to clinical cardiovascular risk factors and echocardiographic left ventricular measures: the Framingham Heart Study.. Circulation.

[10] Garvin P, Nilsson L, Carstensen J, Jonasson L, Kristenson M (2008). Circulating matrix metalloproteinase-9 is associated with cardiovascular risk factors in a middle-aged normal population.. PLoS One.

[11] Cavusoglu E, Ruwende C, Chopra V, Yanamadala S, Eng C (2006). Tissue inhibitor of metalloproteinase-1 (TIMP-1) is an independent predictor of all-cause mortality, cardiac mortality, and myocardial infarction.. Am Heart J.

[12] Lubos E, Schnabel R, Rupprecht HJ, Bickel C, Messow CM (2006). Prognostic value of tissue inhibitor of metalloproteinase-1 for cardiovascular death among patients with cardiovascular disease: results from the AtheroGene study.. Eur Heart J.

[13] Blankenberg S, Rupprecht HJ, Poirier O, Bickel C, Smieja M (2003). Plasma concentrations and genetic variation of matrix metalloproteinase 9 and prognosis of patients with cardiovascular disease.. Circulation.

[14] Ylisirnio S, Hoyhtya M, Turpeenniemi-Hujanen T (2000). Serum matrix metalloproteinases -2, -9 and tissue inhibitors of metalloproteinases -1, -2 in lung cancer–TIMP-1 as a prognostic marker.. Anticancer Res.

[15] Wu ZS, Wu Q, Yang JH, Wang HQ, Ding XD (2008). Prognostic significance of MMP-9 and TIMP-1 serum and tissue expression in breast cancer.. Int J Cancer.

[16] Yukawa N, Yoshikawa T, Akaike M, Sugimasa Y, Rino Y (2007). Impact of plasma tissue inhibitor of matrix metalloproteinase-1 on long-term survival in patients with colorectal cancer.. Oncology.

[17] Yoshikawa T, Cho H, Tsuburaya A, Kobayashi O (2009). Impact of plasma tissue inhibitor of metalloproteinase-1 on long-term survival in patients with gastric cancer.. Gastric Cancer.

[18] Byberg L, Siegbahn A, Berglund L, McKeigue P, Reneland R (1998). Plasminogen activator inhibitor-1 activity is independently related to both insulin sensitivity and serum triglycerides in 70-year-old men.. Arterioscler Thromb Vasc Biol.

[19] Andersson T, Alfredsson L, Kallberg H, Zdravkovic S, Ahlbom A (2005). Calculating measures of biological interaction.. Eur J Epidemiol.

[20] West MJ, Nestel PJ, Kirby AC, Schnabel R, Sullivan D (2008). The value of N-terminal fragment of brain natriuretic peptide and tissue inhibitor of metalloproteinase-1 levels as predictors of cardiovascular outcome in the LIPID study.. Eur Heart J.

[21] Eldrup N, Gronholdt ML, Sillesen H, Nordestgaard BG (2006). Elevated matrix metalloproteinase-9 associated with stroke or cardiovascular death in patients with carotid stenosis.. Circulation.

[22] Tuomainen AM, Nyyssonen K, Laukkanen JA, Tervahartiala T, Tuomainen TP (2007). Serum matrix metalloproteinase-8 concentrations are associated with cardiovascular outcome in men.. Arterioscler Thromb Vasc Biol.

[23] Velagaleti RS, Gona P, Sundstrom J, Larson MG, Siwik D Relations of Biomarkers of Extracellular Matrix Remodeling to Incident Cardiovascular Events and Mortality.. Arterioscler Thromb Vasc Biol.

[24] Frantz S, Stork S, Michels K, Eigenthaler M, Ertl G (2008). Tissue inhibitor of metalloproteinases levels in patients with chronic heart failure: an independent predictor of mortality.. Eur J Heart Fail.

[25] George J, Patal S, Wexler D, Roth A, Sheps D (2005). Circulating matrix metalloproteinase-2 but not matrix metalloproteinase-3, matrix metalloproteinase-9, or tissue inhibitor of metalloproteinase-1 predicts outcome in patients with congestive heart failure.. Am Heart J.

[26] Montaner J, Alvarez-Sabin J, Molina C, Angles A, Abilleira S (2001). Matrix metalloproteinase expression after human cardioembolic stroke: temporal profile and relation to neurological impairment.. Stroke.

[27] Montaner J, Molina CA, Monasterio J, Abilleira S, Arenillas JF (2003). Matrix metalloproteinase-9 pretreatment level predicts intracranial hemorrhagic complications after thrombolysis in human stroke.. Circulation.

[28] Montaner J, Fernandez-Cadenas I, Molina CA, Monasterio J, Arenillas JF (2003). Safety profile of tissue plasminogen activator treatment among stroke patients carrying a common polymorphism (C-1562T) in the promoter region of the matrix metalloproteinase-9 gene.. Stroke.

[29] Castellanos M, Leira R, Serena J, Pumar JM, Lizasoain I (2003). Plasma metalloproteinase-9 concentration predicts hemorrhagic transformation in acute ischemic stroke.. Stroke.

[30] Jefferis BJ, Whincup P, Welsh P, Wannamethee G, Rumley A Prospective study of matrix metalloproteinase-9 and risk of myocardial infarction and stroke in older men and women.. Atherosclerosis.

[31] Rouis M, Adamy C, Duverger N, Lesnik P, Horellou P (1999). Adenovirus-mediated overexpression of tissue inhibitor of metalloproteinase-1 reduces atherosclerotic lesions in apolipoprotein E-deficient mice.. Circulation.

[32] Turunen MP, Puhakka HL, Koponen JK, Hiltunen MO, Rutanen J (2002). Peptide-retargeted adenovirus encoding a tissue inhibitor of metalloproteinase-1 decreases restenosis after intravascular gene transfer.. Mol Ther.

[33] Silence J, Collen D, Lijnen HR (2002). Reduced atherosclerotic plaque but enhanced aneurysm formation in mice with inactivation of the tissue inhibitor of metalloproteinase-1 (TIMP-1) gene.. Circ Res.

[34] Luttun A, Lutgens E, Manderveld A, Maris K, Collen D (2004). Loss of matrix metalloproteinase-9 or matrix metalloproteinase-12 protects apolipoprotein E-deficient mice against atherosclerotic media destruction but differentially affects plaque growth.. Circulation.

[35] Chancey AL, Brower GL, Peterson JT, Janicki JS (2002). Effects of matrix metalloproteinase inhibition on ventricular remodeling due to volume overload.. Circulation.

[36] Spinale FG, Coker ML, Krombach SR, Mukherjee R, Hallak H (1999). Matrix metalloproteinase inhibition during the development of congestive heart failure: effects on left ventricular dimensions and function.. Circ Res.

[37] Creemers EE, Davis JN, Parkhurst AM, Leenders P, Dowdy KB (2003). Deficiency of TIMP-1 exacerbates LV remodeling after myocardial infarction in mice.. Am J Physiol Heart Circ Physiol.

[38] Heymans S, Luttun A, Nuyens D, Theilmeier G, Creemers E (1999). Inhibition of plasminogen activators or matrix metalloproteinases prevents cardiac rupture but impairs therapeutic angiogenesis and causes cardiac failure.. Nat Med.

[39] Rouet-Benzineb P, Buhler JM, Dreyfus P, Delcourt A, Dorent R (1999). Altered balance between matrix gelatinases (MMP-2 and MMP-9) and their tissue inhibitors in human dilated cardiomyopathy: potential role of MMP-9 in myosin-heavy chain degradation.. Eur J Heart Fail.

[40] Hayakawa T, Yamashita K, Tanzawa K, Uchijima E, Iwata K (1992). Growth-promoting activity of tissue inhibitor of metalloproteinases-1 (TIMP-1) for a wide range of cells. A possible new growth factor in serum.. FEBS Lett.

[41] Klein G, Vellenga E, Fraaije MW, Kamps WA, de Bont ES (2004). The possible role of matrix metalloproteinase (MMP)-2 and MMP-9 in cancer, e.g. acute leukemia.. Crit Rev Oncol Hematol.

[42] Opdenakker G, Van den Steen PE, Dubois B, Nelissen I, Van Coillie E (2001). Gelatinase B functions as regulator and effector in leukocyte biology.. J Leukoc Biol.

[43] Jung K (2008). Matrix metalloproteinase-8 and tissue inhibitor of metalloproteinase-1 in serum do not reflect the analytes circulating in blood.. Arterioscler Thromb Vasc Biol.

[44] Rouy D, Ernens I, Jeanty C, Wagner DR (2005). Plasma storage at -80 degrees C does not protect matrix metalloproteinase-9 from degradation.. Anal Biochem.

[45] Dorman G, Kocsis-Szommer K, Spadoni C, Ferdinandy P (2007). MMP inhibitors in cardiac diseases: an update.. Recent Pat Cardiovasc Drug Discov.

