# Supplementary figures and images for: Biomarkers of Extracellular Matrix Metabolism (MMP-9 and TIMP-1) and Risk of Stroke, Myocardial Infarction, and Cause-Specific Mortality: Cohort Study
Source: PLoS One. 2011 Jan 19;6(1):e16185. doi: 10.1371/journal.pone.0016185 (PMC3023803; doi:10.1371/journal.pone.0016185)

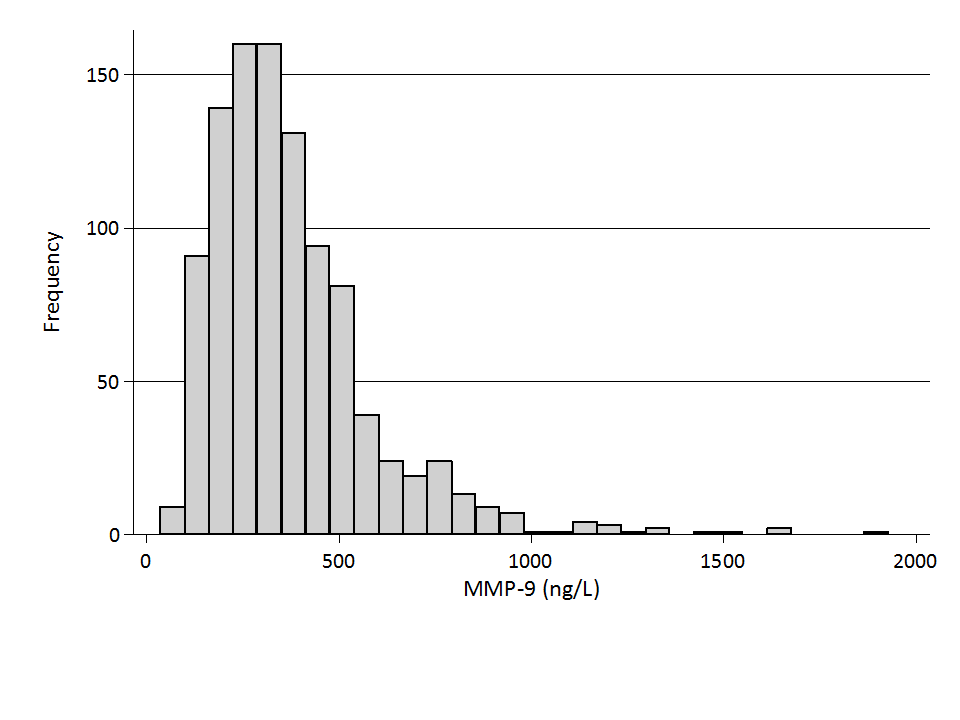

Supplement: Figure S1 — Distribution of s-MMP-9. (TIF) [file pone.0016185.s001.tif]

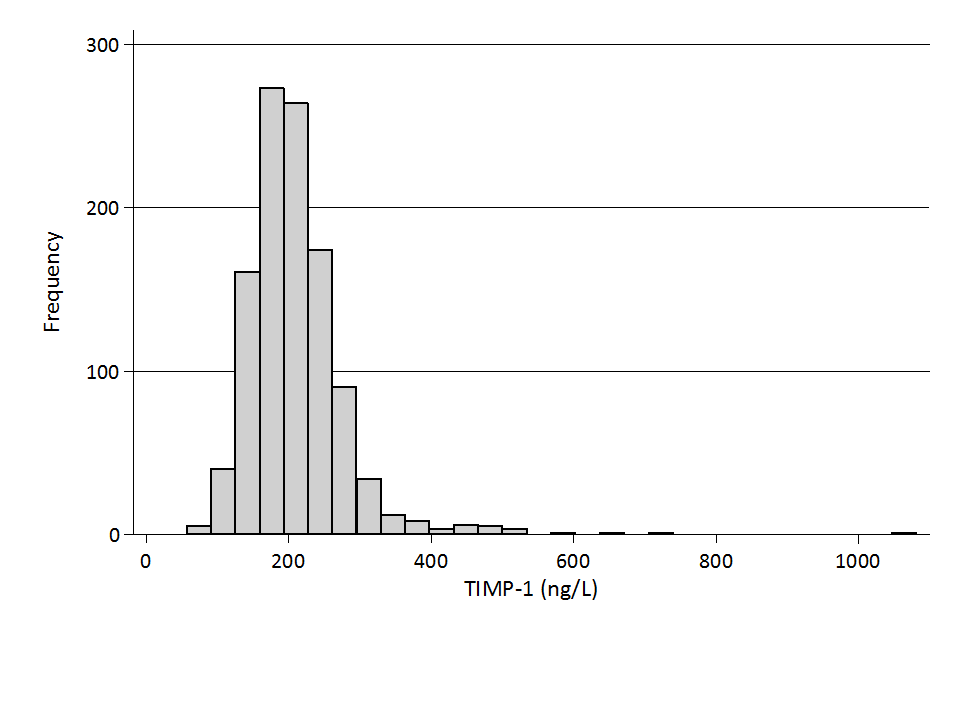

Supplement: Figure S2 — Distribution of s-TIMP-1. (TIF) [file pone.0016185.s002.tif]

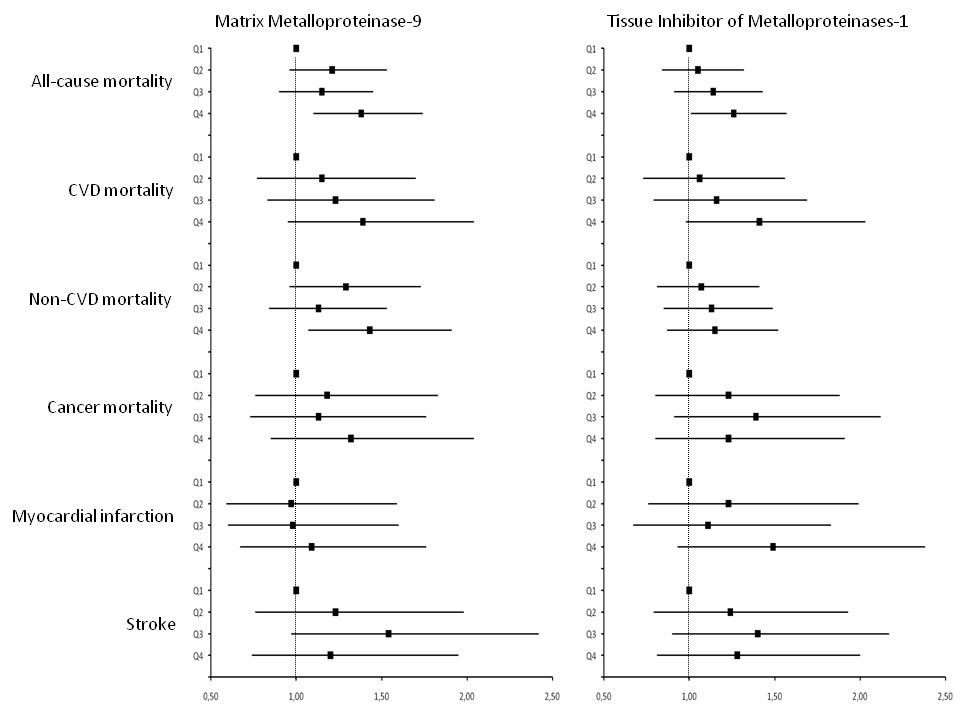

Supplement: Figure S3 — Relations of Quartiles of Matrix Biomarkers to Risk of Cause-Specific Mortality and Cardiovascular Events in the Total Sample. Boxes are Cox proportional hazard ratios, lines are 95% confidence intervals, from models A (adjusted for age and freezer time) for quartiles of matrix biomarkers vs. lowest quartile. Quartile limits for MMP-9 were 228, 332 and 462 ng/L and those for TIMP-1 were 166, 200 and 238 ng/L. (TIF) [file pone.0016185.s003.tif]
