# Supplementary material for: Biomarkers of Extracellular Matrix Metabolism (MMP-9 and TIMP-1) and Risk of Stroke, Myocardial Infarction, and Cause-Specific Mortality: Cohort Study
Source: PLoS One. 2011 Jan 19;6(1):e16185. doi: 10.1371/journal.pone.0016185 (PMC3023803; doi:10.1371/journal.pone.0016185)
Supplement: Table S1 — Relations of Covariates to Risk of Cause-Specific Mortality and Cardiovascular Events in the Total Sample. Data are Cox proportional hazard ratios (95% confidence intervals) from age-adjusted analyses. Associations reported per standard deviation of continuous variables, and for presence vs. absence of dichotomous variables. Previous and current smoking categories compared to never-smokers. (DOC) [file pone.0016185.s004.doc]

|  | All-cause  mortality | | CVD mortality | | Non-CVD-mortality | | Cancer mortality | | Myocardial infarction | | Stroke | |
| --- | --- | --- | --- | --- | --- | --- | --- | --- | --- | --- | --- | --- |
| Systolic blood pressure | 1.12 | (1.04-1.21) | 1.24 | (1.09-1.41) | 1.03 | (0.93-1.14) | 0.95 | (0.82-1.10) | 1.20 | (1.01-1.41) | 1.29 | (1.10-1.50) |
| Antihypertensive drugs | 1.53 | (1.31-1.80) | 2.13 | (1.64-2.76) | 1.24 | (1.01-1.52) | 0.99 | (0.73-1.36) | 1.77 | (1.27-2.48) | 1.69 | (1.24-2.30) |
| Lipid-lowering drugs | 0.91 | (0.69-1.21) | 1.19 | (0.79-1.79) | 0.74 | (0.51-1.09) | 0.66 | (0.36-1.22) | 1.72 | (1.04-2.83) | 1.06 | (0.63-1.79) |
| Diabetes mellitus | 1.36 | (1.11-1.65) | 1.70 | (1.25-2.30) | 1.15 | (0.89-1.49) | 0.88 | (0.58-1.35) | 1.90 | (1.30-2.79) | 1.54 | (1.06-2.24) |
| Body mass index | 1.07 | (0.98-1.16) | 1.16 | (1.02-1.32) | 1.03 | (0.93-1.14) | 1.04 | (0.89-1.21) | 1.08 | (0.91-1.27) | 1.04 | (0.89-1.22) |
| Total cholesterol | 0.98 | (0.91-1.07) | 0.97 | (0.85-1.11) | 1.02 | (0.92-1.12) | 0.95 | (0.81-1.10) | 1.25 | (1.06-1.47) | 1.09 | (0.93-1.28) |
| Glomerular filtration rate | 1.30 | (1.20-1.40) | 1.50 | (1.34-1.68) | 1.10 | (0.99-1.22) | 1.16 | (1.00-1.35) | 1.21 | (1.03-1.42) | 1.21 | (1.03-1.41) |
| C-reactive protein | 1.27 | (1.17-1.38) | 1.38 | (1.21-1.58) | 1.22 | (1.10-1.35) | 1.34 | (1.15-1.57) | 1.28 | (1.08-1.52) | 1.17 | (1.00-1.38) |
| Low previous smoking | 1.29 | (1.04-1.61) | 1.66 | (1.13-2.44) | 1.18 | (0.90-1.54) | 1.24 | (0.84-1.84) | 1.31 | (0.82-2.11) | 0.97 | (0.65-1.47) |
| High previous smoking | 1.48 | (1.17-1.86) | 2.22 | (1.50-3.27) | 1.17 | (0.87-1.56) | 1.06 | (0.68-1.66) | 1.38 | (0.83-2.31) | 1.14 | (0.74-1.75) |
| Current smoking | 1.99 | (1.60-2.48) | 2.42 | (1.65-3.57) | 1.73 | (1.32-2.26) | 1.58 | (1.06-2.38) | 2.18 | (1.38-3.45) | 1.24 | (0.81-1.90) |
